# Two-Dimensional Hole-Array Grating-Coupling-Based Excitation of Bloch Surface Waves for Highly Sensitive Biosensing

**DOI:** 10.1186/s11671-019-3159-8

**Published:** 2019-10-10

**Authors:** Daohan Ge, Jianpei Shi, Ahmed Rezk, Chao Ma, Liqiang Zhang, Ping Yang, Shining Zhu

**Affiliations:** 1 School of Mechanical Engineering, Jiangsu University, Zhenjiang, 212013 People’s Republic of China; 2 National Laboratory of Solid State Microstructures, School of Physics, Nanjing University, Nanjing, 210093 People’s Republic of China; 3 Laboratory of Span-Scale Design and Manufacturing for MEMS/NEMS/OEDS, Jiangsu University, Zhenjiang, 212013 People’s Republic of China; 4 Institute of Intelligent Flexible Mechatronics, Jiangsu University, Zhenjiang, 212013 People’s Republic of China

**Keywords:** 2D grating, Bloch surface wave, Sensitivity, Refractive index sensors

## Abstract

In this study, a surface diffraction two-dimensional (2D) grating structure was placed on the topmost layer of distributed Bragg reflectors (DBRs) for biosensing. Bloch surface wave (BSW) resonance was realized by coupling a 2D subwavelength hole-array grating and could be excited at different locations: the surface of 2D-grating layer or the inter-face between the DBR and bio-solution. Material losses in the multilayer dielectric were measured to test the robustness of this scheme. Both the surface diffraction-grating BSW (DG-BSW) and the alternative guided grating-coupled BSW (GC-BSW) configuration showed markedly enhanced angular sensitivity compared to conventional prism-coupled schematics. Exciting these modes using a grating-coupling technique appears to yield different extreme sensitivity modes with a maximum of 1190°/RIU for DG-BSW and 2255°/RIU for GC-BSW. Refractive index sensors with a high figure of merit may be realized via such compact configurations.

## Background

Specially designed photonic devices represent the possibility of real-time, label-free selective detection of various chemical and biological species for a variety of medical research and environmental monitoring applications and particularly for the optical detection of miniscule quantities of molecules in highly diluted solutions [1–3]. Optical surface mode resonance indices such as surface plasmon polaritons (SPPs) [4–6], microcavity [7], guided-mode resonance [8, 9], and Bloch surface waves (BSW) [10–13] can be utilized to distinguish the generally small modulations of optical parameter(s) reflective of a given biomolecule concentration [14, 15].

The most popular surface wave resonance-based sensing technology is the surface plasmon resonance (SPR) method [4, 16] which works by exciting surface plasmon polaritons along a metal/dielectric interface by incident light. Unfortunately, SPR can only be excited by transverse-magnetic light and absorption accompanied by strong dispersion is inevitable in the metal components. The sensitivity of the SPR biosensors is generally in the order of several hundred nanometers per refractive index unit (nm·RIU^−1^) [17, 18].

BSW is a promising alternative to SPPs. BSW technology based on the low optical loss all-dielectric structure has higher sensitivity and adjustable field enhancement than other surface waves and can be combined with different chemical surface modification methods and optical detection mechanisms [19–21]. Many researchers have experimentally and theoretically demonstrated the superiority of BSW sensors over SPPs sensors [22, 23]. The wavelength sensitivity of 1D-BSW sensors under a Kretschmann configuration is several thousands of nm·RIU^−1^ [24, 25]. Recent researchers [26] demonstrated fiber-based BSW excitation for RI sensing with a sensitivity of about 650 nm/RIU for *p*-polarized light and 930 nm/RIU for *s*-polarized light. Most 1D photonic crystal (1DPC)-based sensors utilize complicated Kretschmann prism-coupled structures to excite BSW. Few researchers have explored grating-coupled-based BSW sensors or other new designs to reduce the complexity of bulk optical components. Vijay et al. [27] did report enhanced sensitivity in a topmost layer grating profile assessed via azimuthal interrogation; the BSW leakage mode is mostly localized inside very narrow grooves which biomolecules do not readily penetrate.

Two-dimensional (2D) grating devices [28–30] have attractive potential as miniature RI sensors due to their large sensing areas and relative ease of fabrication. This paper proposes an alternative excitation scheme based on the 2D grating-coupling mechanism. A BSW is realized on the grating side by depositing air-hole arrays on the surface of a Bragg mirror, which supports BSW on both sides. Here, we present one configuration to simply demonstrate the possibility of coupling a BSW on the tip of the grating-coupled Bragg mirror structure, as well as an alternative scheme which demonstrates the influence of available dielectric loss. We compared the optical performance of sensor configurations for BSW excitation at different locations as discussed in detail below.

## Methods

### Case 1: Surface Diffraction-Grating BSW Configuration (DG-BSW)

**Fig. 1:**
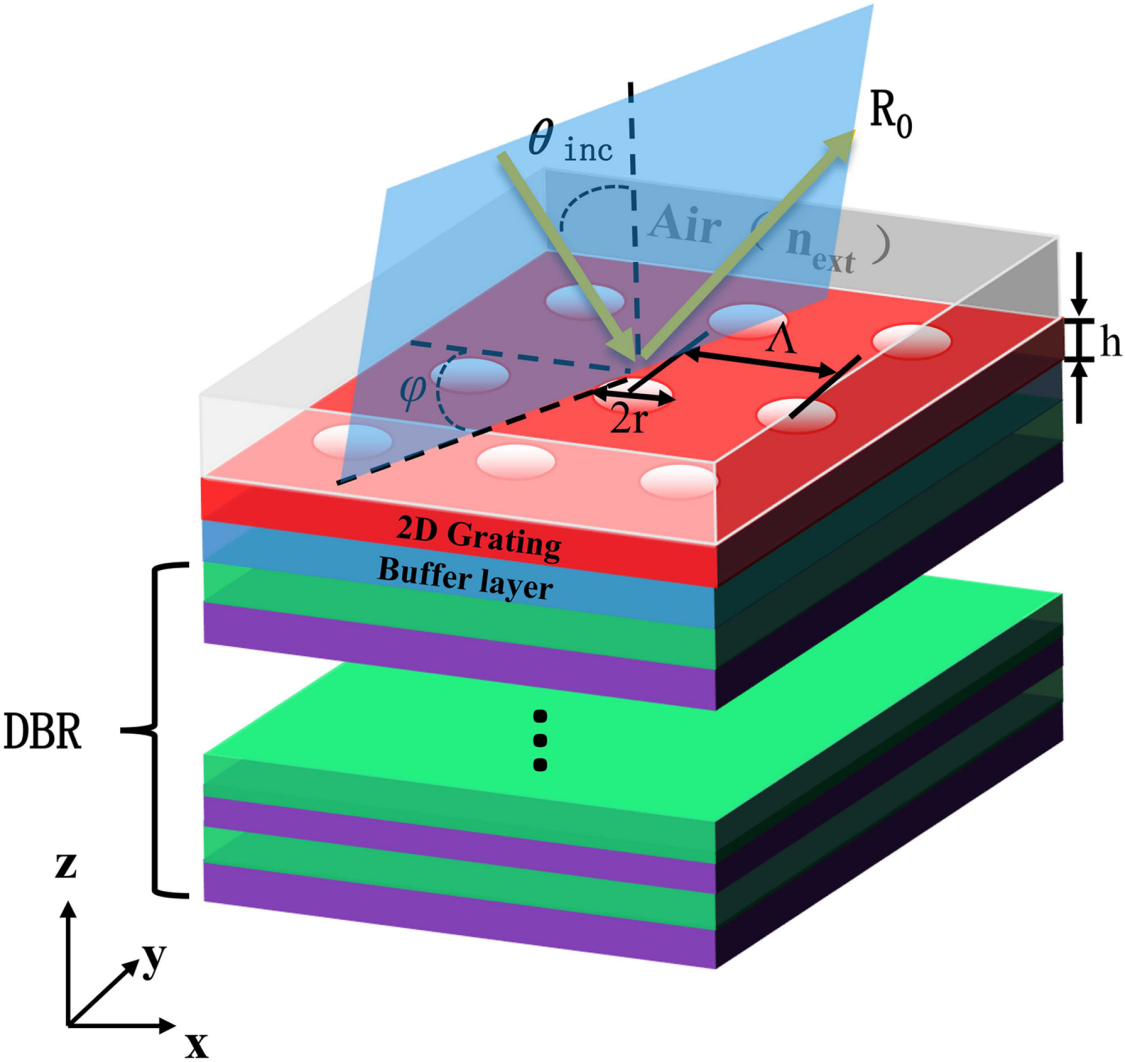
Surface diffraction-grating BSW design in (*x*-*y*-*z*) reference system. Structure includes few-period DBR, buffer layer, and 2D grating. Coupling is mediated by a 2D diffraction grating with period *Λ* = 510 nm, hole radius *r* = 145 nm, and thickness *h* = 116 nm. External medium is assumed as air (*n*_ext_ = 1)

A schematic diagram of the surface diffraction-grating BSW configuration is shown in Fig. 1. The incident angle *θ* (angle between the incident beam and *Z*-axis) and the azimuth angle *φ* (angle between the negative *X*-axis and the incident beam projection in the *x–y* plane) are used to describe the propagation direction of the incident light. In the numerical calculations, we used a five-period DBR (LH)^5^ where the L dielectrics have RI of 1.46 (SiO_2_ at the working wavelength of *λ*_0_ = 657 nm) and the H layers are made of TiO_2_ with the RI of 2.57. The RIs of both TiO_2_ and SiO_2_ over the range of 0.43 to 0.8 μm are expressed as [27]:

1 $$ {n}_{SiO_2}={\left(1+\frac{0.6962{\lambda}^2}{\lambda^2-{0.0684}^2}+\frac{0.4080{\lambda}^2}{\lambda^2-{0.1162}^2}+\frac{0.8975{\lambda}^2}{\lambda^2-{9.8962}^2}\right)}^{\frac{1}{2}} $$

and

2 $$ {n}_{TiO_2}={\left(5.913+\frac{0.2441{\lambda}^2}{\lambda^2-0.0803}\right)}^{\frac{1}{2}} $$

The imaginary parts of the refractive indices refer to the losses in the dielectric layers. These losses include the intrinsic material absorption and the scattering losses in the incident light (${\upgamma}_{{\mathrm{SiO}}_2}=0$ and ${\upgamma}_{{\mathrm{TiO}}_2}={10}^{-4}$, in this work). The DBR can be dimensioned accordingly as a quarter-wavelength stack for an incidence angle at the operational wavelength. The thicknesses of the corresponding layers are respectively *d*_L_ = 100 nm and *d*_H_ = 70 nm.

To fabricate the surface diffraction-grating BSW sensor, a 116-nm-thick layer of silicon nitride (Si_3_N_4_) was deposited on top of the DBR with an air-hole pattern [31, 32] to form the grating layer. A 60 nm-buffer layer which is also made of low refractive index composites (SiO_2_) was inserted between the Bragg mirror and the sub-wavelength hole-array grating. The grating layer is designed to couple the propagating illuminations to the BSW mode. As described above, grating is essentially a 2D periodic array of structural features made from air holes. In the numerical simulations described below, only the physical dimensions of the grating (period *Λ*, hole radius *r*, and thickness *h*) were adjusted to excite BSW under different illumination conditions and to optimize the reflection profiles.

Under the optimized hole-array grating, when BSW is excited, the reflection from the grating-Bragg configuration forms typical Fano resonant profiles [33] with sharp peaks. The locations of the peaks indicate the RI of the region to be probed. The manufacturing process is simple and compatible with existing MEMS manufacturing technologies, which makes the proposed device mass producible and readily integrated into biochips for multiplexed detection at low cost. We conducted the calculations described here with Diffract MOD integrated in RSoft Photonics Suite, which is based on the rigorous coupled-wave analysis (RCWA) method [34, 35] and contains several advanced algorithms with Fourier harmonics that describe periodic dielectric functions.

**Fig. 2:**
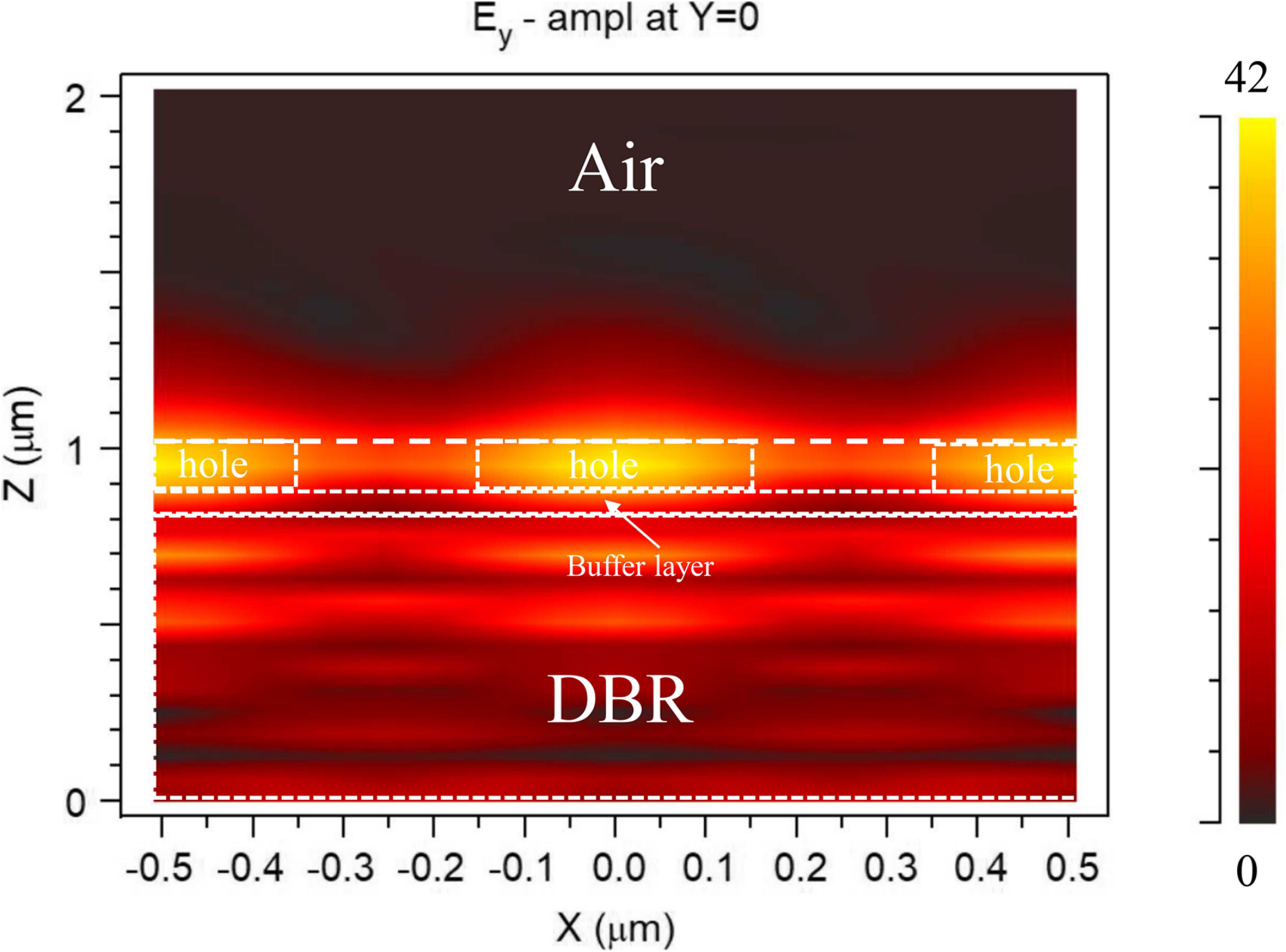
Calculated electric field distribution for *s*-polarized light at resonance where the surface wave is excited on the top surface only. White dotted line represents 2D grating, buffer layer, and DBR layers. Field intensity of BSW mode (yellow region) is concentrated in air holes

Figure 2 shows the simulated electric field distribution for *s*-polarized light when the surrounding RI is 1. The dashed line in Fig. 2 marks the grating–air interface; *z* = 0 is the other side surface of the diffraction-grating BSW sensor. As the figure shows, the electric field is strongly enhanced near the interface and the BSW penetrating depth reaches nearly 200 nm in air. The local field intensity is 42 times the maximum incident light intensity at a polar angle of *θ* = 4.3° and azimuthal angle domain of about *φ* = 12°.

Although the proposed structure can, theoretically, provide BSW excitation in the surface diffraction-grating mode, there are effects related to the detection process which merit careful consideration. As shown in Fig. 2, the strong field is concentrated in the small apertures of the hole-array grating. The analyte in the air cannot easily penetrate into the small-size holes, thus gathering above the grating. The decrease in analyte concentration in the holes brings about a small perturbation in the refractive index, which drives down the detection limit and sensitivity of the BSW sensor. The integration of an incident light illumination device and sensing layer also makes on-chip sensor fabrication difficult; further, it is very difficult to estimate the interaction between them. We explored an alternative configuration to overcome these disadvantages while retaining the exponentially decaying electric field distribution.

### Case 2: Alternative Guided Grating-Coupled BSW Configuration (GC-BSW)

**Fig. 3:**
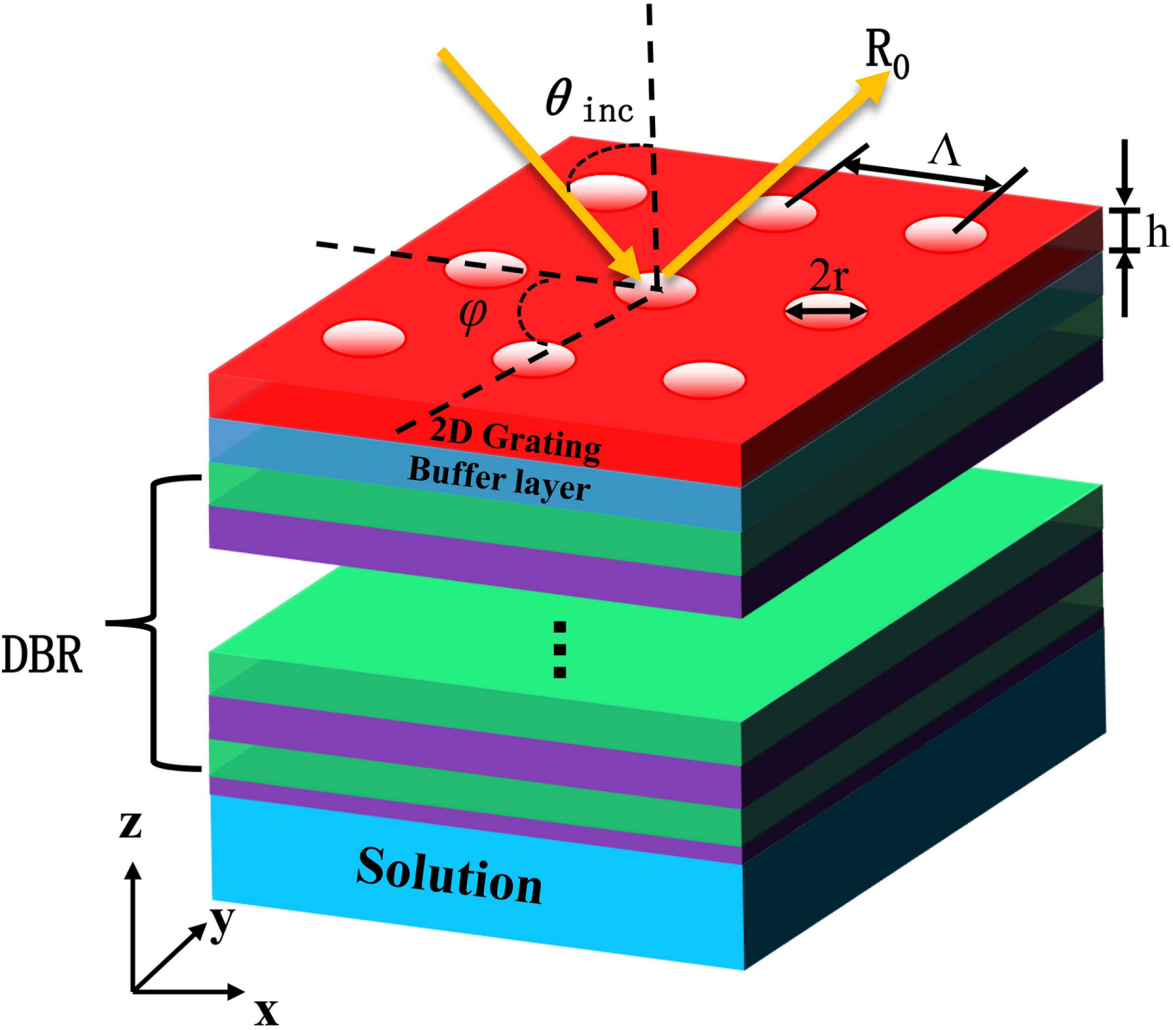
3D schematic diagram of grating-coupled BSW resonance sensor under azimuthal illuminations (*φ*) in (*x*-*y*-*z*) reference system including incident angle (*θ*_inc_), zeroth-order reflection (*R*_0_), and 2D grating parameters (*Λ*, *r*, *h*). Sensing region is located at the bottom of the grating-coupled BSW sensor

In the proposed scheme, the sensing region is now moved to the bottom of the grating-coupled BSW sensor, thereby avoiding any detrimental effects related to surface grating structure penetration (Fig. 3). The materials for the DBR, buffer layer, and grating are similar to those described above. Unlike the DG-BSW sensor, the bottom one TiO_2_ layer thickness was reduced from 70 to 30 nm.

We placed a bio-solution layer with RI near 1.333 (pure water) adjacent to the outmost high refractive index (TiO_2_) layer, where the thickness of the region to be probed is 2 μm. We did not need to precisely control the thickness of the sensing layer in this case, because the outer surface of the probed region does not significantly affect BSW mode excitation. Resonance is formed as *s*-polarized light falls incident on the DBR through the grating at a certain angle, and multiple reflections occur at the bottom defect layer formed by the solution to be tested. The surface defect state structure alters the electromagnetic field distribution on the bottom of the DBR due to surface wave resonance, and multiple reflections in the defect layer form coherent interference. The electromagnetic field is locally enhanced and can fully act on the sample molecules to be tested.

We found that the sensitivity characteristics during dynamic monitoring of the solution to be tested can be improved by the proposed scheme. Similar to SPPs, BSWs are localized at the truncation edge of the 1DPC, at the interface with the external medium. The 2D grating design parameters are the same in the proposed scheme as the previous configuration (DG-BSW): *Λ* = 510 nm, *r* = 145 nm, and *h* = 116 nm. As discussed in detail below, we compared the characteristics of resonant dielectric multi-layer systems DG-BSW and GC-BSW. Our hole-array grating design not only reduces the manufacturing cost, but also provides a relatively fair environment for sensor performance comparison.

## Results and Discussion

**Fig. 4:**
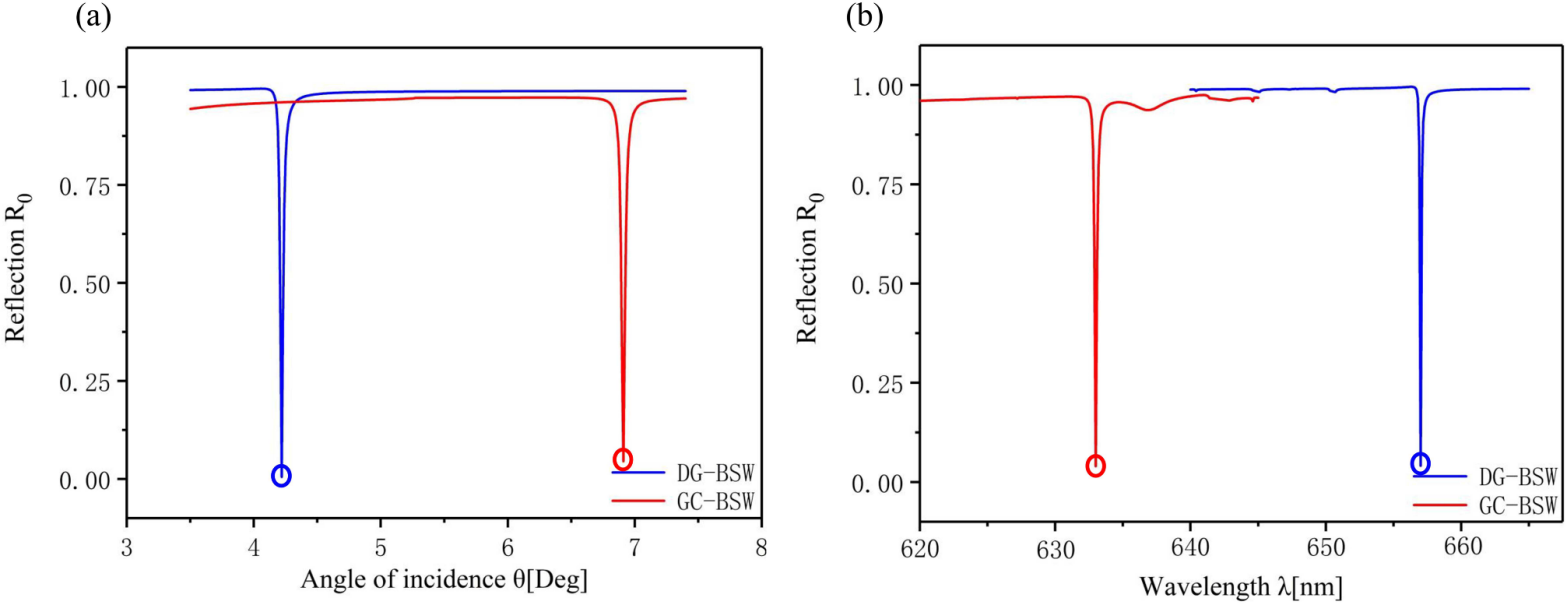
Bloch surface wave at *φ* = 0°. Blue and red curves represent BSW reflectance as a function of incident angle (**a**) and wavelength (**b**) for DG-BSW and GC-BSW configurations, respectively

We designed optimized BSW structures under the two sets of sensing conditions as shown Figs. 1 and 3 with *s*-polarized light in both cases. Reflectivity curves of these modes as a function of angle of incidence and wavelength are shown in Fig. 4a and b, respectively. DG-BSW and GC-BSW cases have sharp resonance features at their excitation both as a function of angle and wavelength. In the DG-BSW device, when the incident wavelength is around 660 nm, a sharp dip peak appeared at *θ* = 4.3° by incident angle interrogation. In the GC-BSW device, the resonant angle *θ* = 7° corresponds to an incident wavelength of 633 nm. We found that although a resonant peak with higher quality factor *Q* (>10^3^) value can be obtained by optimizing the device parameters, the wavelength sensitivity and angle sensitivity of the BSW sensor reached only about 100 nm/RIU and 280°/RIU under non-azimuthal illuminations. Our 3D RCWA simulations are consistent with the literature [24]. We took into consideration the novel design freedom, azimuthal angle *φ*, accordingly.

**Fig. 5:**
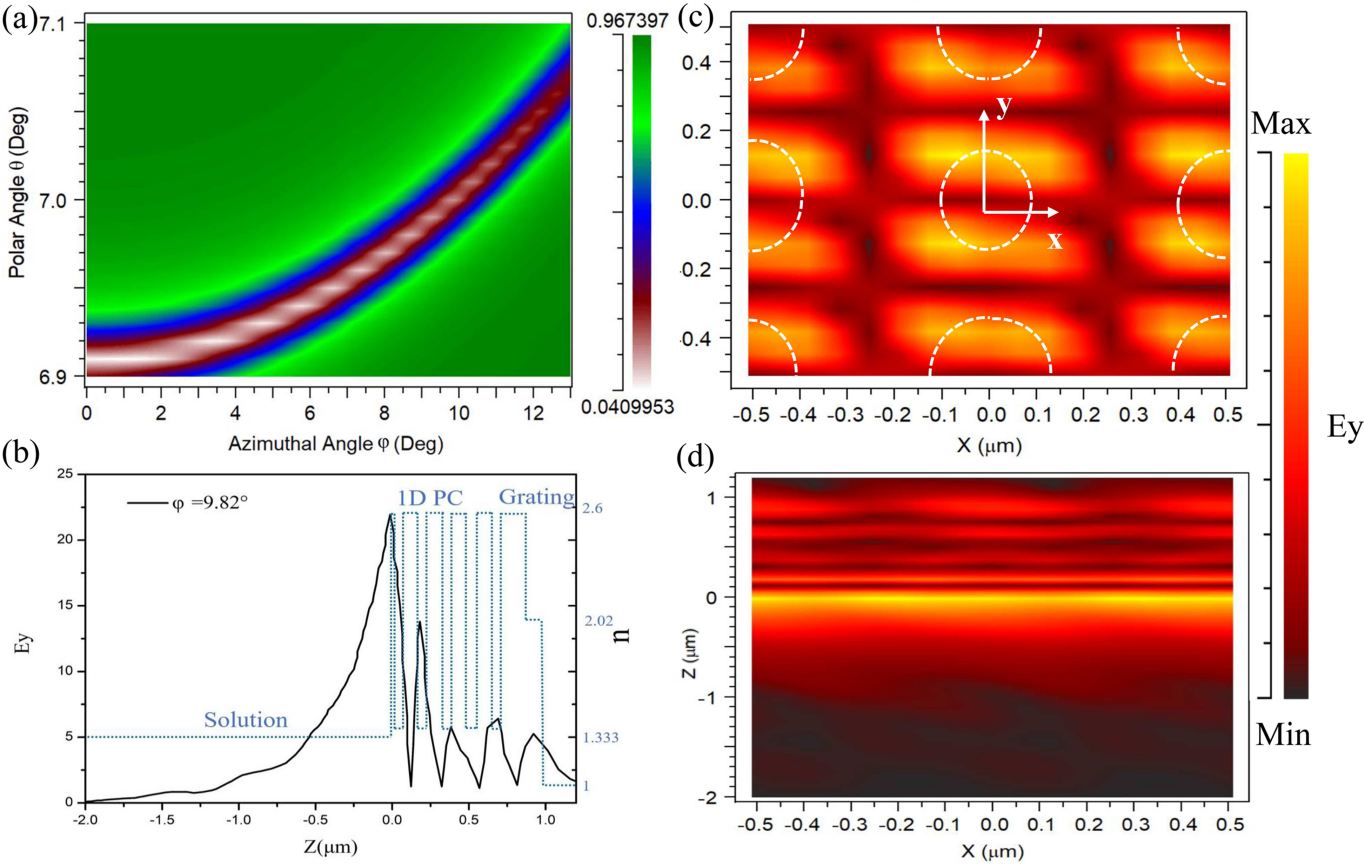
**a** GC-BSW sensor reflection versus azimuthal and polar angles. BSW created by illuminations (*λ*_0_ = 633 nm) near *θ* = 7° and *φ* = 10°. BSW coupling occurs in very narrow areas (white region) with relatively low reflective intensity. **b** Electric field (black line) and refractive index distribution (dark green dotted line) inside sensing configurations (case 2 mode). **c** *x*-*y* **d** *x*-*z* plane views of electric field magnitude map, computed at operational wavelength *λ*_0_ = 633 nm. White dotted line indicates the locations of the holes in the electric field

The simulated reflection of the GC-BSW sensor designed to work near *θ* = 7° and *φ* = 10° is shown in Fig. 5a. BSW coupling occurs in very narrow areas with relatively low reflective intensity (white regions in Fig. 5a). Each polar angle has a corresponding azimuthal angle that satisfies matching conditions to excite the BSW. The BSW mode in the heterostructure decays slowly as polar and azimuthal angles increase, then disappears near *θ* = 7.6° and *φ* = 12°. Considering the difficulty of small angle monitoring, we chose a relatively large angle to couple the BSW. The resonance peak is insensitive to polar angle changes but very sensitive to azimuthal angle changes. We calculated the electric field distribution of the sample point (*θ* = 7°; *φ* = 9.82°) to recognize the resonance (Fig. 5b). Intensity decays toward the grating/air interface and the field oscillated many times throughout the periodic structure and five peaks formed in the L-H refractive index dielectric interface. The light green dotted line in Fig. 5b represents the refractive index distribution of the GC-BSW sensor in the *Z*-axis direction. We found that the magnetic field intensity in the bio-solution gradually decays along the *Z*-direction, as the interaction between light and solution declines with the distance from the truncated layer. The BSW penetration depth reached 2 μm inside the solution, which is tenfold greater than in the DG-BSW configuration.

**Fig. 6:**
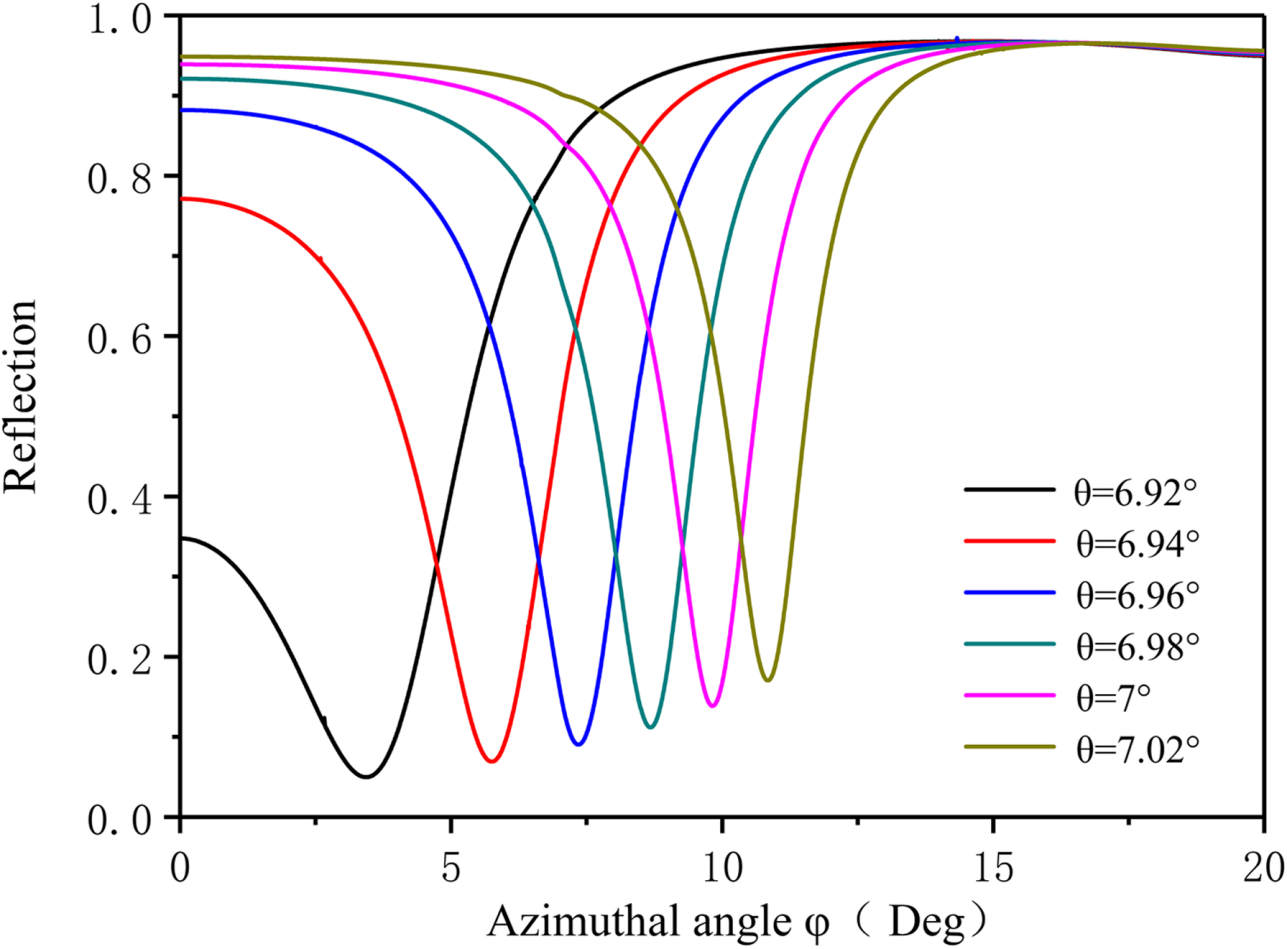
Azimuthal reflectance spectra for different incident angles *θ*. Typical symmetrical line-shapes emerge as azimuthal angle *θ* increases. The BSW resonances shift to higher azimuthal angles due to wave vector matching effect

Figure 5c and d show electric field magnitude maps in *x*-*y* and *x*-*z* planes, respectively, computed at the operational wavelength *λ*_0_ = 633 *nm*. The results of Fig. 5b and d are in close accordance. The field distribution at the solution/TiO_2_ interface largely affects the overall performance of the GC-BSW sensor through the overlap integral between the evanescent field and the spatial distribution of the sensing region’s dielectric constant. We investigated the effects of polar angle on the azimuthal reflection spectra in the GC-BSW configuration by testing polar angles *θ* of 6.92°, 6.94°, 6.96°, 6.98°, 7°, and 7.02°. To assess high sensitivity, we also determined the full width at half maximum (FWHM) of the resonant dip and the dip peak height. As shown in Fig. 6, typical symmetrical line shapes emerged as the azimuthal angle *θ* increased. The resonance peak height increased as resonance peak FWHM decreased. At a larger polar angle, the BSW resonance shifted toward a larger azimuthal angle due to the wave vector matching effect.

Lossless materials (i.e., those with zero values for extinction coefficients κ) are assumed in most numerical simulations [24, 25, 30]. Sinibaldi et al. [36] studied the influence of material losses on the performance of the BSW sensors to find that the extinction coefficient of high index layers κ_H_ only slightly affects the resonance characteristics; they introduced an extinction κ_L_ = 10^−4^ to the low index layers calculated via transfer matrix method (TMM). Lossy materials are necessary to observe a dip in the reflectance spectrum [22].

**Fig. 7:**
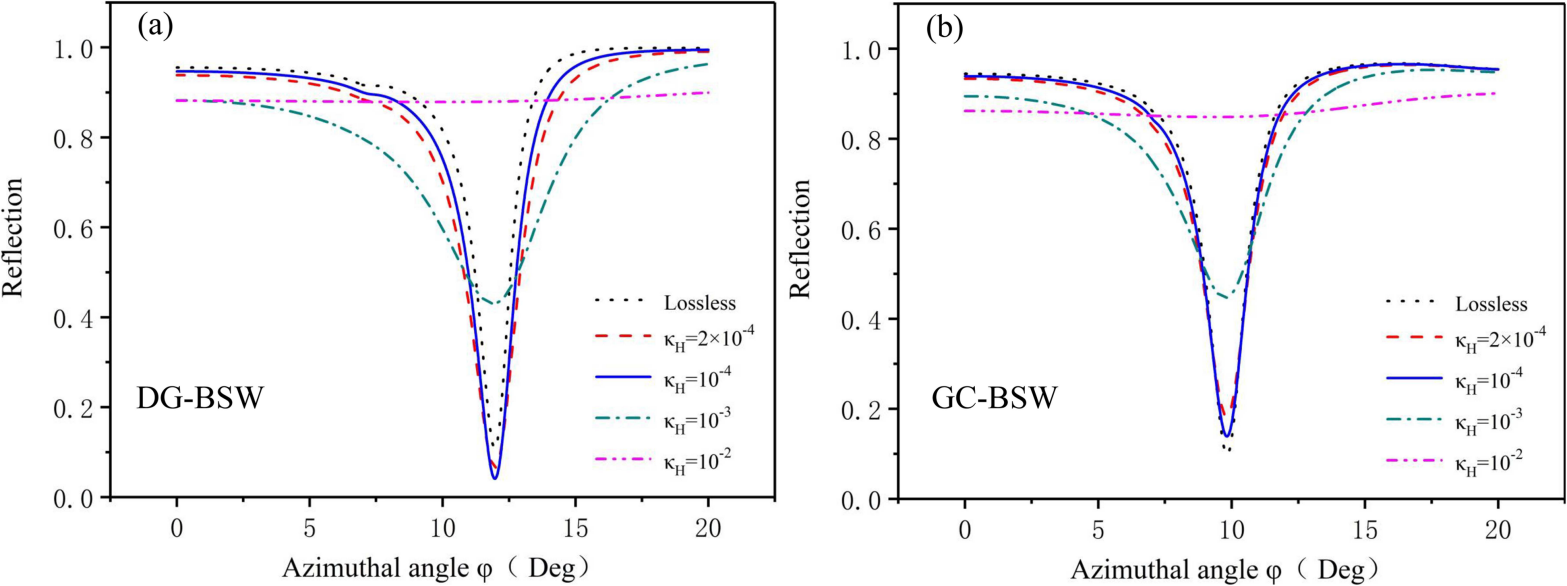
**a** Resonance line shape variations for DG-BSW configuration and extinction coefficients κ_H_ = 0 (lossless), 2 × 10^−4^, 10^−4^, 10^−3^, 10^−2^. **b** Variations for GC-BSW configuration. Lossless TiO_2_ materials excite BSW dip peak in the reflection spectrum. Extinction coefficient values suppress BSW resonance band edge

To study the influence of loss, we assessed the azimuthal reflection spectra from DG-BSW and GC-BSW structures (Figs. 1 and 3) with and without considering the loss as shown in Fig. 7. In our case, lossless TiO_2_ materials can excite the BSW dip peak in the reflection spectrum. Losses in the DBR degrade the BSW line shape obtained in the lossless case. We analyzed the perturbation effect induced by non-zero values for κ on the resonances. In the DG-BSW case, the FWHM of the resonances first decreased and then increased as the extinction coefficient increased from 0 to 10^−3^, while the resonance depth did the opposite. We achieved the optimal BSW resonance line shape when the extinction coefficient κ reached 10^−4^. The resonance quickly dropped off as the coefficients further increased (κ_H_ = 10^−2^). In the GC-BSW configuration, the line width increased slowly as κ_H_ increased as did the BSW resonance peak value. The resonance dip grew wider as energy losses within the biosensor increased.

Our results suggest that lossless TiO_2_ materials yield optimal BSW resonance. When considering loss, an imaginary part as large as 10^−3^ can suppress the amplitude of the reflection and the *Q* of the resonance without affecting the position of the peak. Our simulations also showed that extinction coefficient values play a vital role in determining the optimum compromise between the depth and width (i.e., FWHM) of BSW resonance.

**Fig. 8:**
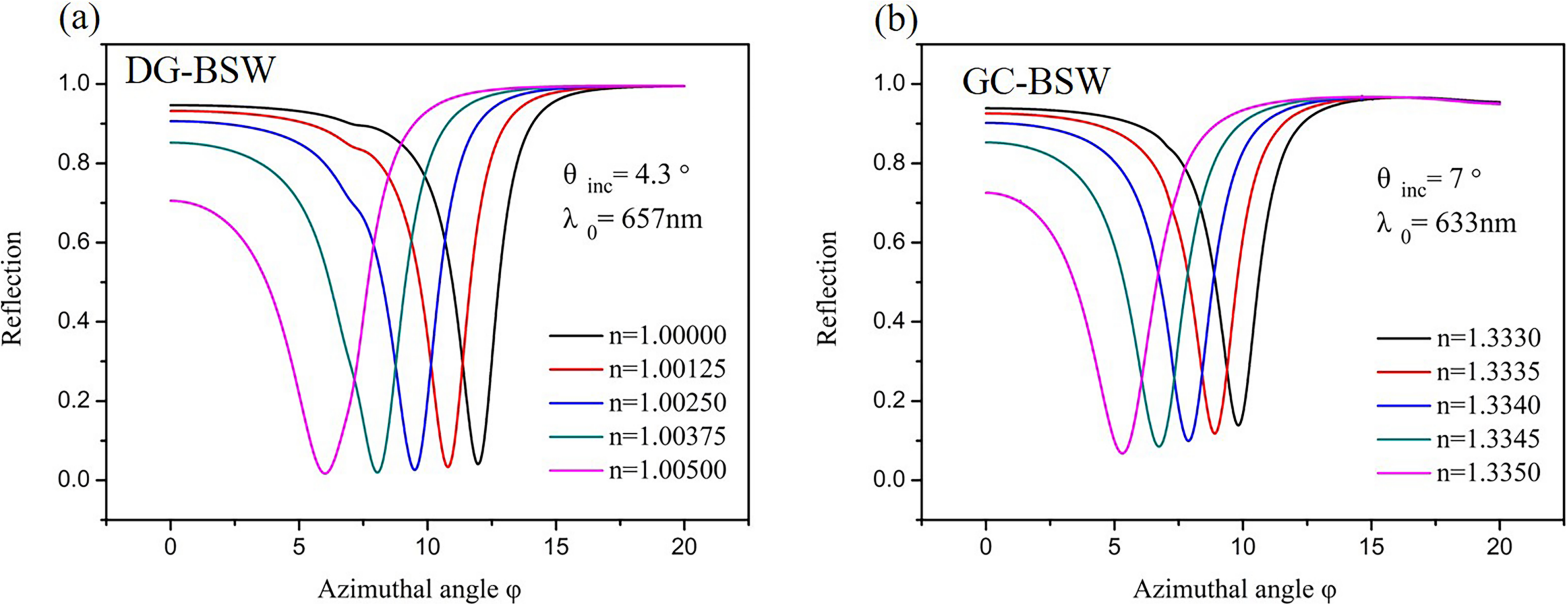
Reflectivity curves as a function of azimuthal angle for different values of solution. **a** DG-BSW configuration, where wavelength (*λ*_0_) and incident angle (*θ*) are fixed at 657 nm and 4.3°; **b** for GC-BSW configuration, *λ*_0_ = 633 nm and *θ* = 7°

The primary goal of this study was to establish a design schematic for label-free sensing platforms based on a 2D grating to excite BSWs, so we continue to explore sensing locations in order to optimize and enhance its performance as a RI sensor. RI biosensors are generally designed to detect small refractive index modulations caused by variations in biomolecule concentration ratios. We thus consider the azimuthal sensitivity (${\mathrm{S}}_{n_{\mathrm{bio}},\varphi }$) a meaningful observable:

3 $$ {\mathrm{S}}_{n_{\mathrm{bio}},\varphi }=\frac{\varDelta \varphi}{\varDelta {n}_{\mathrm{bio}}} $$

where *Δφ* is the change in azimuthal angle and *Δn*_bio_ is the change in sensing layer refractive index. The reflectivity curves as a function of azimuthal angle for different biomolecule values are shown in Fig. 8. For the DG-BSW configuration, the wavelength (*λ*_0_) and incident angle (*θ*) are fixed at 657 nm and 4.3° respectively (Fig. 8a); for the GC-BSW configuration, *λ*_0_ = 633nm and *θ* = 7° (Fig. 8b). When the refractive index of the biomolecules changes evenly, the BSW resonance peaks blue shift in both cases. That is to say, a small change in the refractive index value (*Δn*_bio_ = 0.0005) causes the azimuthal angular shift between the resonance peaks to grow larger at small azimuthal angles.

**Fig. 9:**
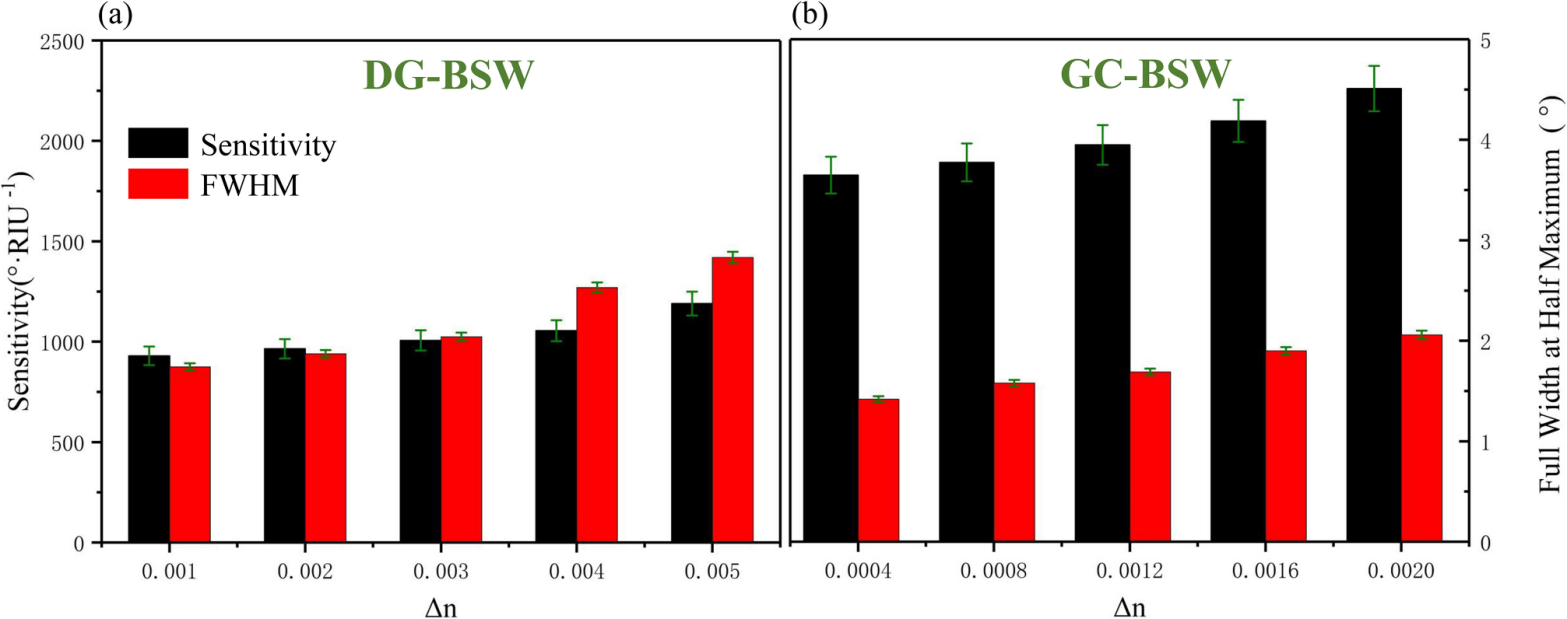
Sensing characteristics of the DG-BSW (**a**) and GC-BSW (**b**) configurations: predicted sensitivity and FWHM as a function of SRI. GC-BSW configuration sensitivity is about twice that of the DG-BSW

We also compared the sensing characteristics of the DG-BSW and GC-BSW configurations to predict sensitivity (black bar) and FWHM (red bar) as shown in Fig. 9 as a function of the surrounding refractive index (SRI). We found that both sensitivity and FWHM monotonically increased as biomolecule variations increased. The sensitivity of the GC-BSW configuration was about twice that of the DG-BSW, while the FWHM of the resonances were narrower in GC-BSW than DG-BSW.

Figure of merit (FOM) [25] is another important sensor performance indicator. FOM can be improved in the RI sensor by decreasing FWHM, increasing the spectral sensitivity S [°/RIU], or both, as FOM∝S/FWHM. The FOM of many optical sensors is constrained by an intrinsic trade-off between spectral sensitivity and FWHM. The azimuthal sensitivity reached 1190°/RIU for the DG-BSW case and 2255°/RIU for the GC-BSW at the maximum extent (Eq. (3)). This implies that the GC-BSW sensor has a closer overlap between the resonant mode and the sensing layer than DG-BSW. The calculations also support the results shown in Figs. 2 and 5b, where the sensing layer of the GC-BSW has a higher light field penetration depth leading to higher sensitivity than DG-BSW.

It is worth noting that the sensitivity of both of the BSW configurations we tested is one order of magnitude higher than that of the conventional prism-based scheme (see Table 1). Unlike any biosensor design based on prism-coupled excitation, there is no strict refractive index limit for the dielectric composite used in DG-BSW or GC-BSW configurations [37–42]. By scaling the parameters of 2D grating and DBR properly, the proposed sensor configurations can be effectively realized in any wavelength range.

**Table 1:** Comparison of layer number, material, and sensitivity of proposed structure with other sensors in literature

No. of layers	Material	Sensitivity	Reference
3	Chalcogenide glasses/SiO_2_ (HLH)	128°/RIU	[24]
4	Ta_2_O_5_/SiO_2_	Experimental = 18°/RIU	[36]
2	Si/SiO_2_	900 nm/RIU or 27.3°/RIU	[37]
21	Porous silicon	72°/RIU or 2038 nm/RIU	[38, 39]
4	TiO_2_/SiO_2_	2255°/RIU	This work

## Conclusions

In this study, we explored surface diffraction 2D grating configurations and sensing applications. We built a multilayer dielectric heterostructure from subwavelength hole-array grating and distributed Bragg reflection (DBR) with few periods (*N* = 5) to realize high-sensitivity BSW resonances with low side-bands. A surface DG-BSW configuration and alternative guided GC-BSW schematic were designed based on RCWA methodology. A theoretical sensitivity of 2255°/RIU was achieved with a small polar angle of the illumination (< 10°) and azimuthal angle sweeps around the same values. Angular sensitivity was one order higher than that of sensors based on prism-coupled polar illuminations (generally not larger than 300°/RIU). The optimized GC-BSW sensor showed a particularly large increase in sensitivity (twofold) and narrower BSW resonance compared to the DG-BSW biosensor. Both 2D-grating-coupled sensor platforms tested in this study show low quality factor compared to traditional BSW RI sensors, but they may be enhanced by tuning the period (*Λ*), hole radius (*r*), and thickness (*h*).

The proposed schemes for exciting Bloch surface waves, DG-BSW and GC-BSW, represent new class compact configurations for highly sensitive biosensing and may afford a valuable opportunity to engineer nanoscale “lab-on-chip” technologies in the future.

## Data Availability

All data generated or analyzed during this study are included in this published article.

## Abbreviations

1DPC: 1D photonic crystal
2D: Two dimensional
BSWs: Bloch surface waves
DBR: Distributed Bragg reflectors
DG-BSW: Diffraction-grating BSW
FOM: Figure of merit
FWHM: Full width at half maximum
GC-BSW: Grating-coupled BSW
Q: Quality factor
RCWA: Rigorous coupled wave analysis
RI: Refractive index
S: Sensitivity
SPPs: Surface plasmon polaritons
SPR: Surface plasmon resonance
TMM: Transfer matrix method
